# Supplementary material for: Circulatory Adipokines and Incretins in Adolescent Idiopathic Scoliosis: A Pilot Study
Source: Children (Basel). 2022 Oct 25;9(11):1619. doi: 10.3390/children9111619 (PMC9688531; doi:10.3390/children9111619)
Supplement: Supplementary file 1 [file children-09-01619-s001.zip › children-1986206-supplementary.pdf]

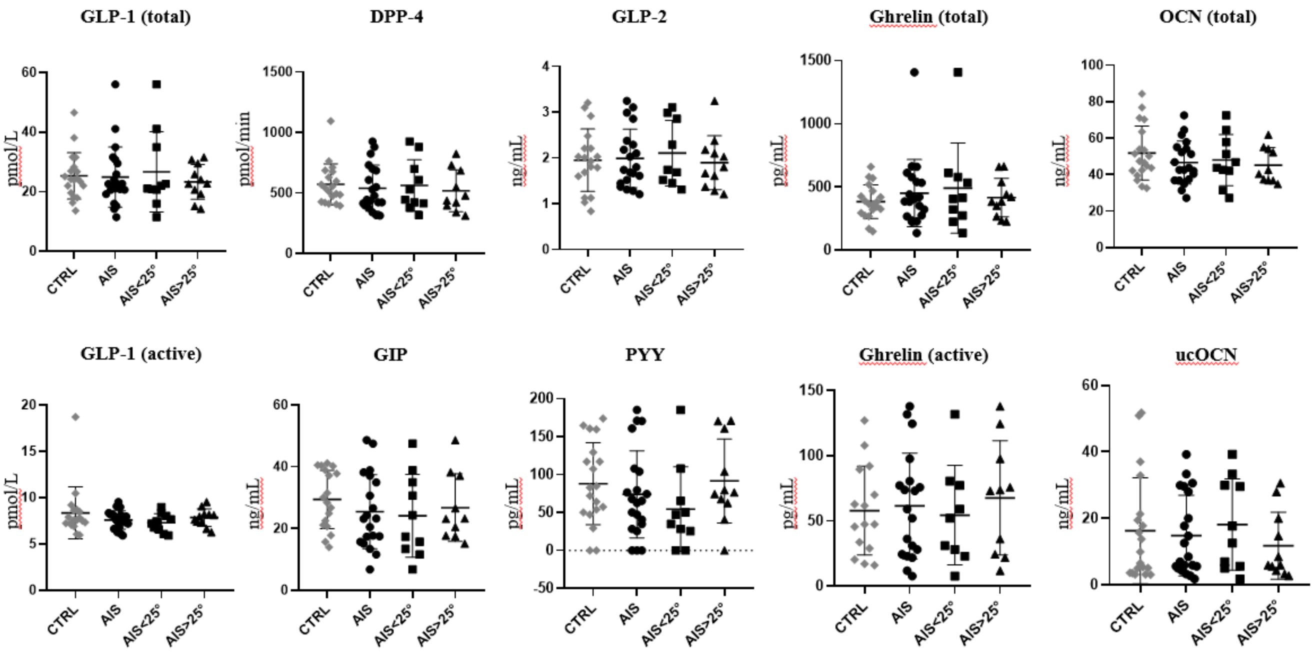

Figure S1. Incretin and osteocalcin levels in control and AIS participants

AIS participants were divided based on curve severity with Cobb angles <25° or >25° as cut-off point. Plasma adipokines levels were assessed by multiplex immunoassays in n=19 CTRL, n=21 AIS, n=10 AIS <25° and n=11 AIS >25°. Student's t-test or Mann-Whitney test. CTRL: Controls; AIS: Adolescent idiopathic scoliosis; GIP: Gastric inhibitory polypeptide; GLP-1: Glucagon-like peptide-1; GLP-2: Glucagon-like peptide-2; PYY: Peptide tyrosine tyrosine; DPP-4: Dipeptidyl peptidase-4; OCN: Osteocalcin; ucOCN: Uncarboxylated osteocalcin.

Table S1. Anthropometry, body composition, BMD and biochemical profile between controls and AIS participants based on curve severity

		Controls (n=19)	AIS < 25° (n=10)	AIS > 25° (n=11)	P value[2]
**Clinical characteristics**	Age (years)	14.8 ± 2.5	14.3 ± 2.0	15.3 ±1.2	0.497
	Highest Cobb angle (°)	-	17.8 ± 3.7	35.6 ± 6.7	**<0.0001***
**Anthropometry**	Height-for-age (z-score)	0.33 ± 1.01	0.60 ± 1.36	0.61 ± 0.98	0.739
	BMI-for-age (z-score)	0.09 ± 1.11	-0.44 ± 0.84	-0.54 ± 0.87	0.185
	waist-to-height ratio	0.47 (0.41-0.56)	0.45 (0.40-0.52)	0.44 (0.40-0.49)	0.072
**Body composition**	Fat (%)	29.2 ± 7.2	25.02 ± 9.1	24.7 ± 4.7	0.193
	Fat mass (g)	15676 ± 6107	12414 ± 6540	12329 ± 3482	0.210
	Lean mass (g)	36558 ± 6466	34912 ± 7010	37292 ± 4780	0.669
**BMD**	Total body less head (z-score)	0.32 ± 0.88[1]	-0.38 ± 0.75	-0.43 ± 0.73	0.033
	Lumbar spine (z-score)	0.00 (-1.40-1.30)[1]	-0.85 (-1.60-1.70)	-0.70 (-1.50-0.60)	0.236
	Femoral neck (z-score)	0.59 ± 1.58[1]	-0.49 ± 1.07	-0.40 ± 1.20	0.079
**Biochemical profile**	Glucose (mmol/L)	4.89 ± 0.32	5.16 ±0.49	4.94 ± 0.28	0.165
	Insulin (pmol/ml)	53.42 ± 21.18	64.41 ± 25.66	51.72 ± 18.99	0.396
	HOMA-IR	1.70 ± 0.74	2.17 ± 1.06	1.64 ± 0.65	0.300
	QUICKI	0.36 ± 0.02	0.35 ± 0.02	0.36 ± 0.03	0.339
	Triglycerides (mmol/L)	0.78 ± 0.24	0.84 ± 0.40	0.81 ± 0.31	0.900
	Cholesterol (mmol/L)	3.76 ± 0.49	4.07 ± 0.59	4.07 ± 0.75	0.313
	HDL-C (mmol/L)	1.35 ± 0.20	1.42 ± 0.31	1.35 ± 0.17	0.655
	LDL-C (mmol/L)	2.31 ± 0.52	2.27 ± 0.31	2.36 ± 0.68	0.258
	ApoB (g/L)	0.63 ± 0.13	0.68 ± 0.09	0.73 ± 0.18	0.197
	ApoA1 (g/L)	1.39 ± 0.18	1.59 ± 0.47	1.40 ± 0.16	0.150
	ApoB/ApoA1	0.46 ± 0.10	0.45 ± 0.09	0.52 ± 0.12	0.248

AIS: Adolescent idiopathic scoliosis; BMD: Bone mineral density.
Data are presented as mean ± SD or median (range).
[1]n=17 controls.
[2]Data were compared using one-way ANOVA or Kruskal-Wallis test for non-normally distributed variables.
*Data were compared using unpaired t-test.

Table S2. Associations between BMI-for-age z-score and metabolic parameters

BMI-for-age (z-score)	Controls		AIS	
	B	P value	B	P value
		**Crude analysis**		
**Adipokines**				
Leptin	0.225	**0.009***	0.160	**0.009***
Adiponectin	-0.009	0.534	-0.003	0.283
Resistin	0.027	0.741	0.009	0.713
Visfatin	-0.027	0.216	-0.003	0.902
**Incretins**				
GLP-1 total	0.034	0.323	-0.006	0.768
GLP-1 active	0.026	0.801	-0.176	0.365
Ghrelin total	-0.003	0.185	-0.001	**0.032***
Ghrelin active	3.511^{E}-5	0.997	-0.009	0.056
GIP	0.018	0.564	-0.023	0.153
GLP-2	0.312	0.456	0.449	0.143
PYY	0.000	0.952	-0.007	**0.033***
DPP-4	0.000	0.765	0.000	0.922
**Insulin metabolism**				
ucOCN	-3.176^{E}-5	0.051	-7.098^{E}-6	0.657
OCN total	-0.037	**0.031***	-0.006	0.747
Insuline	0.025	0.062	0.004	0.612
Glucose	0.596	0.477	-0.573	0.290
HOMA-IR	0.686	0.079	0.020	0.925
QUICKI	-25.802	**0.033***	-4.310	0.554
		**Adjusted model**		
**Adipokines**				
Leptin	0.266	**0.025***	0.161	**0.011***
Adiponectin	-0.007	0.612	-0.004	0.156
Resistin	-0.049	0.584	0.008	0.736
Visfatin	-0.026	0.201	-0.013	0.611
**Incretins**				
GLP-1 total	0.038	0.258	-0.007	0.736
GLP-1 active	0.055	0.587	-0.197	0.334
Ghrelin total	-0.001	0.511	-0.002	**0.032***
Ghrelin active	-0.001	0.896	-0.011	**0.028***
GIP	0.026	0.390	-0.022	0.211
GLP-2	0.236	0.561	0.439	0.228
PYY	-0.001	0.819	-0.007	0.050
DPP-4	0.001	0.799	0.000	0.848
**Insulin metabolism**				
ucOCN	-3.506^{E}-5	0.156	-1.728^{E}-5	0.493
OCN total	-0.044	**0.048***	-0.010	0.703
Insuline	0.026	0.050	0.003	0.713
Glucose	0.753	0.392	-0.624	0.222
HOMA-IR	0.758	**0.045***	-0.001	0.996
QUICKI	-27.893	**0.026***	-3.675	0.624

AIS: Adolescent idiopathic scoliosis; BMI: Body mass index; GLP: Glucagon-like peptide; GIP: Gastric inhibitory peptide; PYY: Peptide tyrosine tyrosine; DPP-4: Dipeptidyl peptidase-4; OCN: Osteocalcin; ucOCN: Uncarboxylated osteocalcin; HOMA-IR: Homeostatic Model Assessment of Insulin Resistance; QUICKI: Quantitative insulin sensitivity check index.
Multiple linear regressions were performed, *$P<0.05$.
Adjusted model for age and energy intake.

Table S3. Associations between fat percentage and metabolic parameters

Fat percentage (%)	Controls		AIS	
	B	P value	B	P value
		**Crude analysis**		
**Adipokines**				
Leptin	1.928	**0.002***	1.478	**0.001***
Adiponectin	0.033	0.767	-0.015	0.488
Resistin	0.431	0.446	-0.050	0.798
Visfatin	-0.101	0.476	-0.139	0.505
**Incretins**				
GLP-1 total	0.043	0.850	0.135	0.394
GLP-1 active	0.658	0.318	-0.713	0.660
Ghrelin total	-0.021	0.168	-0.011	0.054
Ghrelin active	-0.019	0.759	-0.063	0.095
GIP	-0.081	0.699	-0.086	0.532
GLP-2	0.691	0.803	2.525	0.309
PYY	-0.005	0.887	-0.040	0.137
DPP-4	0.004	0.746	-0.009	0.295
**Insulin metabolism**				
ucOCN	0.000	0.099	0.000	0.191
OCN total	-0.186	0.124	-0.184	0.188
Insuline	0.097	0.294	0.076	0.316
Glucose	-3.726	0.528	-5.609	0.151
HOMA-IR	2.239	0.402	1.018	0.603
QUICKI	-104.558	0.220	-57.178	0.400
		**Adjusted model**		
**Adipokines**				
Leptin	2.109	**0.001***	1.424	**0.002***
Adiponectin	-0.042	0.680	-0.018	0.430
Resistin	0.048	0.930	-0.035	0.855
Visfatin	-0.123	0.333	-0.257	0.170
**Incretins**				
GLP-1 total	0.034	0.866	0.108	0.484
GLP-1 active	0.732	0.189	-0.316	0.843
Ghrelin total	-0.010	0.505	-0.009	0.173
Ghrelin active	-0.022	0.708	-0.069	0.095
GIP	0.024	0.903	-0.130	0.339
GLP-2	-0.236	0.922	4.562	0.087
PYY	-0.027	0.404	-0.033	0.240
DPP-4	-0.003	0.868	0.011	0.465
**Insulin metabolism**				
ucOCN	0.000	**0.034***	1.982^{E}-5	0.919
OCN total	-0.292	**0.024***	-0.020	0.917
Insuline	0.116	0.141	0.054	0.495
Glucose	0.927	0.873	-4.013	0.309
HOMA-IR	3.386	0.138	0.639	0.748
QUICKI	-134.534	0.069	-41.941	0.546

AIS: Adolescent idiopathic scoliosis; BMI: Body mass index; GLP: Glucagon-like peptide; GIP: Gastric inhibitory peptide; PYY: Peptide tyrosine tyrosine; DPP-4: Dipeptidyl peptidase-4; OCN: Osteocalcin; ucOCN: Uncarboxylated osteocalcin; HOMA-IR: Homeostatic Model Assessment of Insulin Resistance; QUICKI: Quantitative insulin sensitivity check index.
Multiple linear regression were performed, *$P<0.05$.
Adjusted model for age and energy intake.

Table S4. Associations between total body BMD z-score and metabolic parameters

BMD total body (z-score)	Controls		AIS	
	B	P value	B	P value
		**Crude analysis**		
**Adipokines**				
Leptin	0.036	0.683	0.072	0.240
Adiponectin	0.000	0.992	-0.001	0.758
Resistin	0.038	0.582	0.024	0.224
Visfatin	0.006	0.723	0.004	0.830
**Incretins**				
GLP-1 total	0.020	0.478	-0.027	0.097
GLP-1 active	0.014	0.868	-0.047	0.781
Ghrelin total	0.001	0.511	0.000	0.728
Ghrelin active	-0.001	0.900	0.000	0.955
GIP	0.002	0.932	-0.021	0.138
GLP-2	0.010	0.977	0.208	0.446
PYY	-0.002	0.704	-0.001	0.623
DPP-4	-0.002	0.236	0.001	0.182
**Insulin metabolism**				
ucOCN	$-1.537^{E}-5$	0.268	$9.434^{E}-6$	0.493
OCN total	-0.018	0.223	0.007	0.650
Insuline	0.007	0.589	-0.004	0.610
Glucose	0.322	0.659	-0.207	0.620
HOMA-IR	0.204	0.563	-0.125	0.535
QUICKI	-11.504	0.307	0.214	0.976
		**Adjusted model**		
**Adipokines**				
Leptin	-0.329	**0.020***	-0.021	0.819
Adiponectin	0.004	0.815	-0.001	0.603
Resistin	-0.019	0.831	0.018	0.231
Visfatin	0.011	0.687	0.031	0.320
**Incretins**				
GLP-1 total	-0.003	0.927	-0.011	0.459
GLP-1 active	0.085	0.389	0.008	0.954
Ghrelin total	0.002	0.406	0.000	0.602
Ghrelin active	0.004	0.634	-0.004	0.459
GIP	-0.002	0.937	-0.026	**0.026***
GLP-2	-0.137	0.717	0.029	0.906
PYY	-0.002	0.600	-0.001	0.815
DPP-4	-0.001	0.610	-0.001	0.414
**Insulin metabolism**				
ucOCN	$-4.466^{E}-5$	0.058	$-2.281^{E}-5$	0.125
OCN total	-0.026	0.213	-0.022	0.163
Insuline	0.001	0.911	-0.002	0.777
Glucose	0.447	0.601	-0.428	0.283
HOMA-IR	0.039	0.904	-0.081	0.664
QUICKI	-5.920	0.590	0.762	0.909

BMD: Bone mineral density; AIS: Adolescent idiopathic scoliosis; BMI: Body mass index;
GLP: Glucagon-like peptide; GIP: Gastric inhibitory peptide; PYY: Peptide tyrosine tyrosine;
DPP-4: Dipeptidyl peptidase-4; OCN: Osteocalcin; ucOCN: Uncarboxylated osteocalcin;
HOMA-IR: Homeostatic Model Assessment of Insulin Resistance;
QUICKI: Quantitative insulin sensitivity check index.
Multiple linear regressions were performed, *P<0.05.
Adjusted model for age, fat mass, height-for-age, calcium, vitamin D and physical activity.
